# Molecular characterization of precise *in vivo* targeted gene integration in human cells using AAVHSC15

**DOI:** 10.1371/journal.pone.0233373

**Published:** 2020-05-26

**Authors:** Huei-Mei Chen, Rachel Resendes, Azita Ghodssi, Danielle Sookiasian, Michael Tian, Serena Dollive, Laura Adamson-Small, Nancy Avila, Cagdas Tazearslan, John F. Thompson, Jeff L. Ellsworth, Omar Francone, Albert Seymour, Jason B. Wright

**Affiliations:** Homology Medicines Inc., Bedford, Massachusetts, United States of America; University of Florida, UNITED STATES

## Abstract

Targeted gene integration via precise homologous recombination (HR)-based gene editing has the potential to correct genetic diseases. AAV (adeno-associated virus) can mediate nuclease-free gene integration at a disease-causing locus. Therapeutic application of AAV gene integration requires quantitative molecular characterization of the edited sequence that overcome technical obstacles such as excess episomal vector genomes and lengthy homology arms. Here we describe a novel molecular methodology that utilizes quantitative next-generation sequencing to characterize AAV-mediated targeted insertion and detects the presence of unintended mutations. The methods described here quantify targeted insertion and query the entirety of the target locus for the presence of insertions, deletions, single nucleotide variants (SNVs) and integration of viral components such as inverted terminal repeats (ITR). Using a humanized liver murine model, we demonstrate that hematopoietic stem-cell derived AAVHSC15 mediates *in vivo* targeted gene integration into human chromosome 12 at the *PAH* (phenylalanine hydroxylase) locus at 6% frequency, with no sign of co-incident random mutations at or above a lower limit of detection of 0.5% and no ITR sequences at the integration sites. Furthermore, analysis of heterozygous variants across the targeted locus using the methods described shows a pattern of strand cross-over, supportive of an HR mechanism of gene integration with similar efficiencies across two different haplotypes. Rapid advances in the application of AAV-mediated nuclease-free target integration, or gene editing, as a new therapeutic modality requires precise understanding of the efficiency and the nature of the changes being introduced to the target genome at the molecular level. This work provides a framework to be applied to homologous recombination gene editing platforms for assessment of introduced and natural sequence variation across a target site.

## Background

AAV-mediated targeted gene integration is a powerful method for the durable expression of a gene in cells and tissues. Integration of a gene payload into the genome can be accomplished through multiple DNA damage response pathways. The two most common mechanisms of gene integration are homologous recombination (HR) and non-homologous end joining (NHEJ) [1–3]. HR is initiated by a cross-over between two homologous sequences, whose seamless resolution leaves no unintended mutation at the target locus [4, 5]. NHEJ joins two ends of a broken DNA, often leaving repair scars such as insertions and deletions at the site of repair [6, 7].

Gene editing technologies are rapidly being adapted to correct disease-causing mutations at the DNA level and have the therapeutic potential to treat a broad range of monogenic diseases [8–16]. Achieving targeted gene integration without introducing additional mutations is critical in establishing the therapeutic viability of a gene editing platform aimed at introducing corrective changes including full cDNA sequences that result in the expression of a therapeutic protein.

Targeted gene integration can be broadly divided into two categories: one involves the use of endonucleases such as TALEN, zinc finger nucleases or CRISPR-Cas9 with repair DNA templates that use NHEJ as the predominant pathway for integration [17]. The other category is nuclease-free such as AAV-mediated gene integration using the recombinant AAV genome as the repair template. While the precise integration pathway is still debatable, HR is one of the proposed pathways [1, 2, 18–21]. AAV-mediated integration has been reported to be free of random mutations at the target locus, supportive of HR as the underlying DNA repair mechanism [5].

AAVs are increasingly being used to deliver DNA repair templates in combination with endonucleases for therapeutic development [22]. A recent paper that coupled AAV with CRISPR-cas9 achieved high level of gene integration [23]. However, the authors also reported high levels of viral ITR sequences at the CRISPR-cas9 generated integration sites. It is uncertain if integration of ITR sequences would cause any adverse effect on human cells [24], suggesting a systematic evaluation should be carried out for different therapeutic gene integration designs.

Here, we designed AAVHSC15, an AAV vector derived from human hematopoietic stem cells, to perform gene integration at the human phenylalanine hydroxylase (*PAH*) locus. *PAH* mutations result in an inborn error of metabolism of the phenylalanine pathway [25], resulting in the disease phenylketonuria or PKU (OMIM #261600). PKU is an autosomal-recessive, monogenic rare disease caused by bi-allelic loss-of function mutations in the *PAH* gene. Targeted integration of an intact *PAH* cDNA into the native locus in the liver of PKU patients at sufficient efficiency and fidelity has great potential for long-term disease correction. Such potential is demonstrated in a recent study in the *Pah*^*enu2/enu2*^ mouse model [26]. The *Pah*^*enu2/enu2*^ model contains a single missense mutation in the *PAH* gene associated with PKU disease phenotypes. Correction of this missense mutation using CRISPR/cas9-induced DNA breaks coupled with a donor DNA template containing the correct nucleotide at the enu2 missense mutation reduced serum phenylalanine in the treated animals demonstrating that gene editing is a promising therapy for PKU [26].

AAVHSC15 has significant hepatocyte tropism as demonstrated through the I.V. administration into non-human primates using a dose of 7x10^13^ vg/kg (viral genome per kilogram [27]. Furthermore, AAVHSC15 was recently shown to transduce mouse hepatocytes at levels high enough to correct the disease phenotype of PKU [28]. To determine if AAVHSC15 transduces and edits human hepatocytes, we selected a chimeric human-murine liver model generated from FRG mice (Fah^-/-^/ Rag2^-/-^/Il2rg^-/-^). This model is a surrogate system that allows experimentation with functional human hepatocytes in a physiological environment. The humanized mouse liver system has been used to evaluate clinically relevant AAVs [29] and to improve AAV capsid selection [30]. The main goal of this study is to understand the on-target integration efficiency and fidelity following *in vivo* editing of the *PAH* locus in a humanized liver (HuLiv) mouse model at the DNA level.

There are two major challenges in characterizing AAV-mediated integration frequency and fidelity. First, AAV viral genome repair templates include lengthy homology arms flanking a payload sequence, so complete characterization requires sequence coverage spanning multiple kilobases. Second, careful experimental design and quality control is required to prevent unintegrated viral genomes from convoluting editing measurements and characterization. To quantify the frequency of gene integration and sequence fidelity at a disease relevant gene, we used AAVHSC15 vectors to insert a full-length *PAH* cDNA into the 1^st^ intron of the human *PAH* gene in a humanized liver mouse model. To characterize AAV-mediated gene integration in this model, we developed a next-generation sequencing approach that provides estimates for integration efficiencies in human hepatocytes *in vivo*. Furthermore, we evaluated three widely used sequence variant analysis tools for use in the detection and quantitation of mutations across the targeted integration site. Additionally, we employed long-read sequencing techniques to query for viral ITR integrations and other alterations at the target site.

## Materials and methods

### Vector production and quantification

Recombinant AAV vectors were produced by triple transfection of HEK293 cells with a plasmid containing the hPAH vector genome, a second plasmid containing either the AAVHSC15 capsid sequences and AAV2 Rep gene, and a third plasmid containing the adenovirus helper genes. At 72 hours post-transfection, cells were separated from supernatant by centrifugation and lysed for 1 hour in a buffer containing Tris-HCl, NaCl, triton, magnesium chloride, and benzonase. Cell debris was clarified by centrifugation and purified using an AAV9 affinity resin. Vector was enriched for full capsids by cesium ultracentrifugation and buffer exchanged and formulated in DPBS pH 7.3 containing 35 mM NaCl, 1.0% w/v sucrose and 0.03% w/v poloxamer 188. Vectors were titered by quantitative PCR using primers targeting the SV40 region. All vectors were analyzed by silver- and Coomassie Blue-stained SDS-PAGE for VP1, 2 and 3 ratios, endotoxin (<10 EU/mL) and capsid content by ELISA.

### FRG^®^ mice study

*FRG*^®^ mice HuLiv model were generated by Yecuris Corporation (www.yecuris.com) with human hepatocytes from donor HHM19027. Animal experiments were evaluated and approved by the animal ethics committee at Yecuris, following ethical guidelines established by the American Veterinary Medical Association. AAVHSC15-mediated integration of the human *PAH* locus in FRG mice was assessed at Yecuris Corporation. A single hepatocyte donor was used for each set of experiments. To reduce the contribution of mouse hepatocytes, two mice on study were removed from CuRx nitisinone (NTBC) for > 25 days and from CuRx SMX/TMP antibiotic for > 3 days prior to dosing. No adverse events were observed. Each of the two mice was anesthetized with isoflurane and received 1 x 10^14^ vg/kg of AAVHSC15-hPAH by dosing via the retro-orbital sinus (Day 0). One day post-dose, mice were put on CuRx NTCB and continued with the standard NTCB water cycle until study termination. Six weeks post-dose, mice were anesthetized with Mouse Cocktail (Yecuris PN00289) and immediately perfused via the portal vessel with collagenase in Perfusion Solution II (1mL 5x Collagenase/4mL of Perfusion Solution II). A sample of dissociated liver, prior to separation of human from murine hepatocytes (5 x 10^5^ cells), was collected, centrifuged at 100*g* at 4°C and resuspended in Trizol. Human and mouse hepatocytes were filtered through 100μm and 60μm Millipore filters and purified using Miltenyi AutoMACs. Post-purification, 1.5 x 10^5^ cells from the starting material and purified human and mouse hepatocytes were plated on collagen-coated plates for purity assessment by immunocytochemistry using a rabbit anti-human FAH and rat anti-mouse OC5G10 antibodies. Samples were analyzed for human *PAH* and *GAPDH* relative to mouse *Pah* and *Gapdh* with genomic DNA isolated from each sample. Purity of human hepatocytes was >99% for all samples while mouse preparations contained between 2–20% human hepatocytes. Cell pellets were either immediately placed in Trizol or flash frozen in liquid N_2_ and stored at -80°C.

### Target integration nested PCR

Genomic DNA was extracted from hepatocytes using the Qiagen DNeasy kit (Cat# 69504) according to the manufacturer’s protocol and quantitated with the Qubit dsDNS HS kit (Invitrogen, Cat# Q32854). A positive control plasmid was constructed for the nested PCR experiments. This included the human PAH payload, the flanking homology arms, and 500bp of human genomic sequence to the left and right of the homology arms. For a negative control, 100 ng of human genomic DNA from Clontech was used. 100ng genomic DNA from HuLiv mice was used for PCR. PrimeStar Max DNA polymerase master mix (Takara, Cat# R045B) were used for the PCR reaction. Primer sequences are listed in S1 Table.

### Linkage ddPCR for integration frequency

To measure editing efficiency, a droplet-digital linkage assay was employed using two primer and probe sets, one targeting the PAH payload and a second targeting the human or mouse genomic sequence, respectively. Each set of positive/negative controls and sample DNA was assayed with FAM and HEX probes resulting in each droplet producing one of four possible signals; empty, FAM-positive (payload alone); HEX-positive (genomic alone); and HEX+FAM positive (contains payload and genomic sequence). Genetic linkage was measured by determining the proportion of partitioned droplets containing both the payload and genomic sequence relative to the expected frequency given independent distribution of each species of DNA as described in [16, 31]. Primer and probe sequences are listed in S1 Table.

### 3-primer NGS for integration frequency

A 3-primer approach was used to calculate editing efficiency on both left and right integration sites of the *PAH* editing construct. The two amplicons share the outward primer located on the genomic DNA flanking the homology arm, while the inward primers were unique for unedited alleles or for the edited alleles. Primers and amplicons size for left or right integration sites. Primer sequences are listed in S1 Table.

Amplicon size: Left unedited: 1360bp, Left edited: 1364bp, Right unedited: 1263bp, Right edited:1316bp.

To correct sampling bias and the differences in amplicon efficiency of WT and integrated amplicons, a 5-step control panel for both left and right integration sites was prepared for downstream data correction. The control panels consisted of WT amplicon and integrated amplicon at 1pg/μl that included 0%, 1%, 2%, 5%, and 10% integrated amplicon. 1pg of each control panel and 100ng of genomic DNA samples from transduced hepatocytes were PCR-amplified with high fidelity polymerase PrimeStar Max (Takara R045B) with 0.25μM of three Left primers or three Right primers in a 20μl reaction. PCR cycling conditions used were: 98°C 10 sec, 20 cycles for control panels or 32 cycles for testing genomic DNA samples (98°C 10sec, 56°C 10sec, 72°C 30sec). Genomic DNA purchased from Clontech (Cat # 636401) was used as the untreated sample.

PCR products were column purified (Zymo Research D4013) and size selected for 1200bp-1400bp with Blue Pippin 1.5% cassette (SageScience BDF1510). The size-selected amplicons were quantitated with Qubit4 DNA HS (ThermoFisher Q32854), and 1ng of amplicons was used for preparation of the sequencing library with a Nextera Library Prep Kit (Illumina #FC-131-1024). The Illumina DNA library preparation protocol was used (Document #15031942v03). MiSeq Reagent V3 150 Kit (Illumina MS-102-3001) was used to perform paired-end sequencing, 75nt in each direction, with a MiSeq System (Illumina SY-410-1003).

Editing efficiency calculations:

1. **Demultiplexing**: Sequencing reads were first demultiplexed and adaptor trimmed into individual samples with the MiSeq on board processing pipeline.
2. **QC**: Sequencing reads were checked with FastQC [32] for sequencing quality.
3. **Integrated and WT allele counts**: Reads that contained the 12 base junction at the homology arm to unedited specific sequence (6 bases at the homology arm, and 6 bases at the unedited specific sequence) were counted as unedited reads. Similarly, reads that contained the 12 base junction at the homology arm to edited specific sequences were counted as edited reads. The “Observed” % of editing was calculated as (edited read counts / total read counts *100%).
4. **Correction with standard curve**: The “Observed” % editing against “Expected” % editing for the 5-step control (0% to 10% edited) was plotted and a linear correction formula was computed to fit the observed data points to the expected data points. The correction formula was applied to the human genomic DNA sample and the corrected editing efficiency calculated. A commercially available, human genomic DNA was used as a negative control. The negative control showed a low level of integration (0.46% on the left site and 0.17% on the right site). The low integration level suggests a potential contamination during sample preparation and represents the noise level of this assay.

### Fidelity analysis

**Fig 1:**
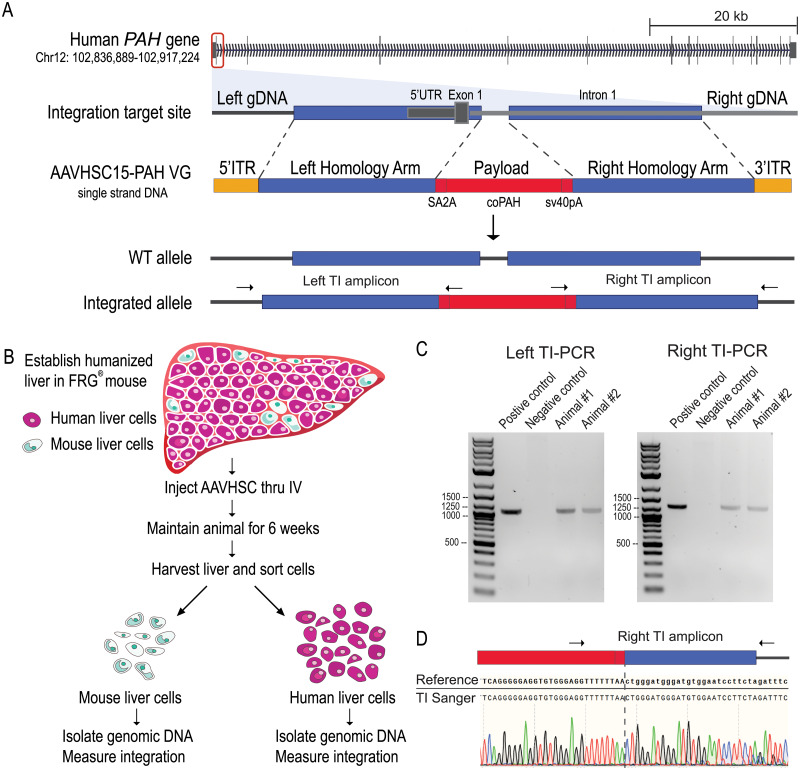
hPAH viral genome design and FRG^®^ mouse model. A. Human *PAH* on Chr12. hPAH viral genome targets intron 1 of *PAH*. The payload consists of a SA (splicing acceptor), T2A element, codon-optimized *PAH* cDNA, and SV40 late polyadenylation sequence. The left and right homology arms, 960bp and 911bp respectively, flank the payload. Both ends of the viral genome are capped with AAV2 ITRs. After vector treatment, a fraction of the alleles had payload integration and the rest of the alleles remained WT. B. FRG^®^ mouse model. The treated mouse liver contained ~20% mouse liver cells and ~80% human liver cells derived from a single donor. Vector was injected retro-orbitally at 1 x 10^14^ vg/kg dosage. Animals were maintained for 6 weeks post-dosing. The liver was harvested and liver cells were separated by species. Mouse and human liver genomic DNA were used for characterization. HuLiv image adapted from Yecuris^®^ with permission. C. Target integration PCR (TI PCR) was used to detect integration events. The arrows on the integrated allele in Fig 1A indicate the approximate primer locations for both left and right integration sites. D. Sanger sequence of right TI PCR amplicon. 50bp region around the payload to right homology arms are shown.

To determine if editing was accompanied by *de novo* mutations at the integration sites, we sequenced both left and right homology arms of both integrated alleles and WT alleles from the vector treated animals. Molecule number of WT alleles and integrated alleles in the input DNA samples were roughly matched to reduce PCR bias: 200ng of genomic DNA was used to amplify edited alleles, and 20ng gDNA for WT alleles. PCR mixture: high fidelity polymerase PrimeStar Max (Takara R045B) with 0.25μM of each forward and reverse primer in a 20μl reaction. PCR cycling condition: 98°C 10sec, 35 cycles for edited alleles or 32 cycles for unedited alleles (98°C 10sec, 56°C 10sec, 72°C 30sec), and final 72°C 30sec. WT alleles were amplified with one step PCR with the following primers, and size-selected for 1000-1500bp with Blue Pippin (Sage science, 1.5% gel cassette). Primer sequences are listed in S1 Table. Integrated alleles were PCR amplified with two-step nested PCR with the same primers listed in the Target integration section (S1 Table, under Fig 1 TI PCR section). The amplicons from the first step PCR were size-selected for 1000-1500bp with Blue Pippin and used for nested PCR. These amplicons were purified, made into a Nextera library, and sequenced with a MiSeq v3 kit as described above.

To understand the detection limit of the assay, we built an amplicon-based control panel by mixing two right homologous arm amplicons, different by one base (a T to G variation), at 0%, 0.1%, 0.5%, 1%, 2%, 5%, 10%, 50, and 100%. The amplicon control panel went through the same sequencing steps as testing samples.

Variant sequencing analysis:

1. **Demultiplex and QC**: as described above.
2. **Read Quality trim**: Fastq reads passed the cut-off >Q30 were retained for analysis.
3. **Mapping**: The paired-end sequences were mapped to the homology arm reference using BWA-MEM (version 0.7.17). The reads from the mapped bam file that fail to map as a proper pair were discarded.
4. **Variant detection on control panel**: Variants were called with GATK-Mutect2 (version 4.1.3.0), Illumina-Pisces (version 5.2.11.163), and LoFreq (version 2.1.3.1). In Mutect2, variants were filtered using the option–min-base-quality-score 30. In Pisces, variants were filtered using options -MinVF 0.0005 -MinBQ 30 -MinDepth 200. In LoFreq, the use of base-alignment quality was disabled using the option -B. The three variant callers were compared using the ground truth. Pisces and LoFreq performed equally well reporting true positives; however, Pisces had a higher precision at 0.5% threshold (lower number of false positives), compared to Mutect2 and LoFreq.
5. **Variant detection on HuLiv samples**: The variant analysis on HuLiv genomic samples was performed using Pisces as described above. Sequencing coverage for almost all positions was above 10,000x. We considered a variant to be a *de novo* variant with the following criteria: (a) at >0.5% variant frequency and passed filters, (b) appeared in both technical sequencing duplicates, and (c) not observed in WT alleles.

### ITR integration analysis

WT and integrated amplicons used for fidelity analysis, described above, were made into long-read Oxford Nanopore sequencing libraries following the manufacturer’s protocol (NBE_9065-v109) and sequenced with a MinION. To satisfy the high input DNA requirement for Nanopore library preparation, WT amplicons from animal 1 and 2 were pooled, and integrated amplicon from animal 1 and 2 were pooled. A control amplicon that represents ITR integration at the left homology arm was also sequenced.

Oxford Nanopore ITR analysis.

1. **Demultiplex**: Guppy barcoder was used to trim nanopore adaptor sequences and demultiplexing samples.
2. **Read quality trim and remove short reads**: Fastq reads were filtered by read quality > 90 and read length over 400nt using Seqkit [33].
3. **Second read length trim**: The expected size for WT and integrated amplicon falls within 1200-1400bp without ITR. Reads that were shorter than 1000nt or longer than 2000nt were removed.
4. **Mapping**: A concatenated reference containing (a) ITR integrated control amplicon sequence, (b) seamless integration amplicon sequence and (c) WT amplicon sequence, was made for both left and right integration sites. Reads were mapped to the concatenated reference with minialign (https://github.com/ocxtal/minialign) default setting.
5. **Clean sample cross contamination**: A small fraction of sample cross-contamination was observed from the first mapping result. This potentially happened at the sample preparation step or at the Nanopore demultiplexing step. The unique regions of each sample (left control amplicon was longer than left edited amplicon, WT amplicon contained WT specific sequence) was used to remove cross-contamination.
6. **Remapping**: Reads were mapped to the concatenated reference again. 84 reads from the left integrated sample and 51 reads from the right integrated sample mapped to the ITR containing reference.
7. **Examine reads with partial ITR**: We consider that a read contains the ITR based on the following criteria: (a) full length: covers a portion of the payload and the entire homology arm, (b) contains at least 5 nucleotides matching the payload facing ITR sequences [6], and (b) contains genomic sequence outside of the integrated junction.
  Examine the 84 ITR containing reads from the left site:
    74 reads primed with only the reverse primer, 4 reads were mis-priming events, 3 reads were too short to identify the origin, and none of these 81 reads had genomic sequence outside of the integrated junction. The other 3 reads carried left control specific SNPs that escaped the first round of the cleaning process.
  Examine the 51 reads from the right site:
    All 51 reads were not full length, and none had genomic sequence outside of the integrated junction.
8. **Count read and display**:
  Mapped bam files were used to count read number and were converted to bigwig files to show full coverage. Fifty randomly selected reads and coverage files were displayed using Integrated Genome Viewer.

## Results

### Integration of human *PAH* cDNA into the endogenous *PAH* locus via AAVHSC15 in the HuLiv mouse model

To understand the nature of AAVHSC-mediated gene integration, we designed a single-stranded AAV-editing vector as shown in Fig 1A. The payload was composed of a codon-optimized *PAH* cDNA with a splice acceptor (SA) and ribosome-skipping element (T2A) at the 5’ end and an SV40 late polyadenylation signal at the 3’ end. The left (5’) and right (3’) homology arms, 960bp and 911bp respectively, flanked the payload. Both ends of the viral genome are capped with AAV2 ITRs. When integration occurs, the payload is inserted into *PAH* intron 1, and a 140bp SINE (short interspersed nuclear element) repetitive sequence in intron 1 is replaced. Following transduction, we anticipated a fraction of the human genomes would carry the *PAH* payload integration (hereafter termed integrated alleles), and the rest would be unmodified wild-type alleles. The viral genome was packaged into AAVHSC15 capsids (see Materials and methods) and referred to as “hPAH vector”. AAVHSC15 was selected due to its high tissue tropism towards liver [27].

We characterized hPAH (human PAH) vector-targeted integration in as physiologically relevant a system as possible, the FRG^®^ humanized mouse model (Fig 1B) which has a humanized liver. When treated as described here, the FRG^®^ mouse livers are composed of ~80% human hepatocytes from a single human donor and ~20% remaining mouse hepatocytes [34]. This model enables *in vivo* characterization of targeted integration outcomes in functioning human liver cells. While not a completely human system, this mouse model is as close as we could come to a human model. Two mice were treated with a single dose of hPAH vector via retro-orbital injection (1 x 10^14^ vg/kg) to maximize the transduction of hepatocytes in order to facilitate the characterization of on-target gene integration events. Six weeks post-dosing, the livers were harvested, and human and mouse hepatocytes were separated. Mouse and human hepatocyte genomic DNA were isolated and characterized separately. Many viral genomes per host haploid genome were detected in human and in mouse genomic DNA samples, indicating the vector transduced hepatocytes from both species. There was higher transduction in human hepatocytes (>200 viral genome per human haploid genome) relative to mouse hepatocytes (~35 viral genome per mouse haploid genome). These measurements are based on viral genome copy number relative to target genome copy number. Notably, murine hepatocytes are frequently tetraploid and octoploid, resulting in the potential underestimation of murine transduction measurements.

To determine whether targeted integration occurred at the endogenous *PAH* locus, we performed a target integration PCR (TI-PCR, also referred as “In-Out” PCR) with one primer in the payload and a second primer in the genomic DNA outside the homology arm, on both left and right integration sites (Fig 1A). Both human liver samples produced positive TI amplicons of the expected size (Fig 1C). Importantly, no TI amplicon was observed from isolated human genomic DNA mixed with a high copy of viral genomes. This control supports the concept that the TI amplicon in treated human cells is not an artifact of rearrangement between human and viral genomes during the PCR reaction. The TI amplicon was purified and Sanger sequenced. The resulting sequencing data matched with reference, which reflected seamless integration events at the limit of detection of this method (Fig 1D, showing Right TI amplicon Sanger sequencing trace). Although end-point TI PCR indicates the presence of edited alleles, it does not provide quantitative information or detection of low-level insertions or deletions at the genomic junctions.

### Integration frequency measurement in human hepatocytes is comparable using two quantitative methods

**Fig 2:**
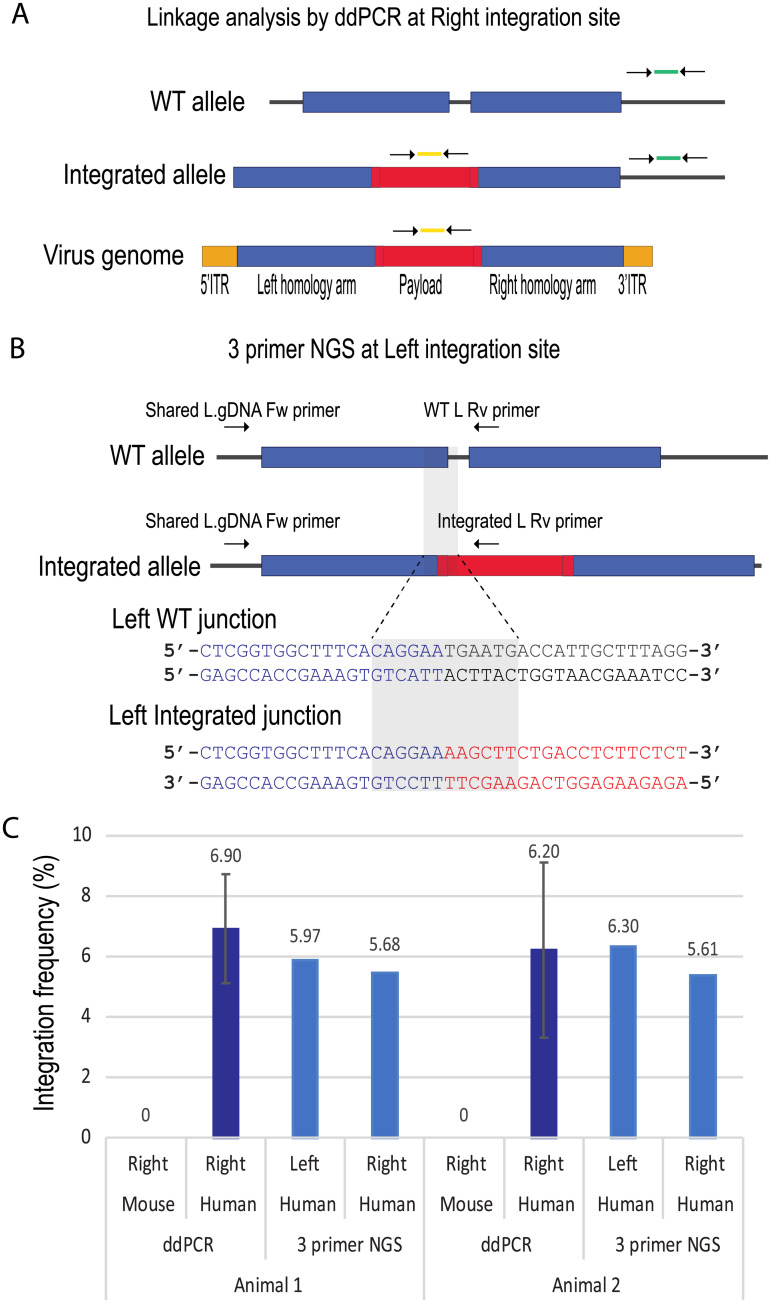
Integration frequency reaches 6% in human hepatocytes by two quantitation assays. A. Schematic of linkage two-color ddPCR. Two primer-probe sets were used in this assay. FAM primer probe set (in yellow) detected coPAH payload, and HEX (in green) detected human genomic DNA outside of right homology arm. B. Schematic of 3 primer NGS assay at the left integration site. Three primers were used to amplify WT integration amplicon and integrated amplicon in a single PCR reaction. PCR products were sequenced with Illumina’s platform. The 12 base sequences unique for WT junction or for integrated junction are highlighted. The same assay was designed and performed at the right integration site. C. Linkage ddPCR and 3 primer NGS results of human genomic DNA or mouse genomic DNA. Note that a mouse specific HEX primer probe set was used for the mouse samples. In ddPCR, each sample was analyzed 3 times, and the error bar represents standard deviation. In 3 primer NGS, both left and right integration sites were queried.

To measure hPAH vector integration frequency, we developed two quantitation assays: linkage analysis by droplet digital PCR (ddPCR) at the right integration site and 3-primer NGS at both left and right integration sites. Linkage ddPCR utilizes two color primer-probe sets, one detecting the payload in the FAM channel, and the other detecting genomic DNA outside the right homology arm in the HEX channel (Fig 2A). Sample DNA is partitioned into nanoliter scale oil droplets at DNA input concentrations in which the majority of droplets contain a single copy of a target DNA strand. Upon partitioning, each is analyzed for fluorescence emission from end-point PCR in each of two channels of probes. FAM and HEX are specific to the payload and target genome, respectively. Four types of droplets were tallied: FAM^-^/HEX^-^: empty droplet; FAM^+^/HEX^-^: payload only (viral genome or sheared integrated alleles); FAM^-^/HEX^+^: right genomic DNA only (WT alleles or sheared integrated alleles); and FAM^+^/HEX^+^: integrated alleles, or a droplet contains viral genome and WT sequences by chance (false positive). The molecular counting of each species is calculated by fitting the raw data counts to a Poisson distribution to adjust for the probability of multiple strands of target sequence per droplet. Genetic linkage was calculated by determining the proportion of droplets containing both the payload and genomic sequence relative to the expected frequency given an independent distribution of each species of DNA. Both human and mouse genomic DNA were subjected to separate linkage ddPCR assays with species-specific genomic DNA primer-probe sets. No measurable integration was observed in mouse *Pah* genomic DNA. The human and mouse *PAH* genes share 66.4% sequence identity over non-gapped regions, suggesting that this level of similarity is not sufficient to drive cross-species targeted integration events. In contrast, between 6.2% and 6.9% integration was observed in the human *PAH* locus in both animals (Fig 2C).

Our second 3-primer NGS approach does not assume linkage of two locations on the integrated alleles. This assay uses three primers, one common genomic DNA primer paired with WT and integration specific primers, respectively, in a single PCR reaction. The resulting amplicons are used to capture the sequence junctions for both the WT and integrated alleles (Fig 2B illustrates the left integration site). The PCR products were sequenced using the Illumina MiSeq platform. Data were analyzed using a counting method that tallies sequence reads that contain a specific 12 nucleotide sequence spanning genotype-specific junctions between the homology arm and either the WT sequence or the integrated payload. Both sequences include 6 bases of the homology arm. The WT sequence includes 6 bases of the naturally occurring repetitive sequence while the integrated sequence includes 6 bases of the payload. A critical step of this assay is to normalize the PCR efficiencies between the WT and integrated amplicons. We generated 5-step control standard samples composed of 0%, 1%, 2%, 5% and 10% integrated alleles. These control samples were 3-primer amplified and sequenced using the same procedure as the treated human genomic DNA samples. Both left and right standard curves showed a strong linear correlation between expected and observed measurements. Differences in PCR efficiencies for both left- and right-side assays were used to correct for amplicon bias of each sample (S1 Fig Left side, R^2^ = 0.99, Pearson correlation p-value < 0.0001; Right side, R^2^ = 0.99, Pearson correlation p-value = 0.0022). The linear relationship suggests that, between 0–10% integration frequency, the differences between the two amplicons PCR efficiency is predictable and the 5-step control standards can be used for normalization. We performed 3-primer NGS on both left and right integration sites on each human liver genomic DNA. In animal 1, we observed 6.0% integration frequency on the left side and 5.7% on the right side. The level of integration in animal 2 was similar to animal 1, 6.3% on the left and 5.6% on the right.

The advantage of linkage ddPCR is that it provides a binary readout of integration and is minimally affected by PCR efficiency. However, it does not provide base pair level sequence information and has a limited range of detection (0.25–500 copies/ul). The 3-primer NGS approach provides a quantitative measurement at the sequence level spanning both the right and left homology arms and is insensitive to excess viral genomes in the sample but requires precisely controlled molar concentrations of standard controls. Taken together, the editing frequency measured by two orthogonal methods shows integration of the codon-optimized *PAH* into intron 1 in the human *PAH* locus are comparable, average at 6.2% (+/-0.5%) for animal 1 and 6.0% (+/-0.3%) for animal 2.

### Integration is precise without *de novo* mutations across the homology arms

**Fig 3:**
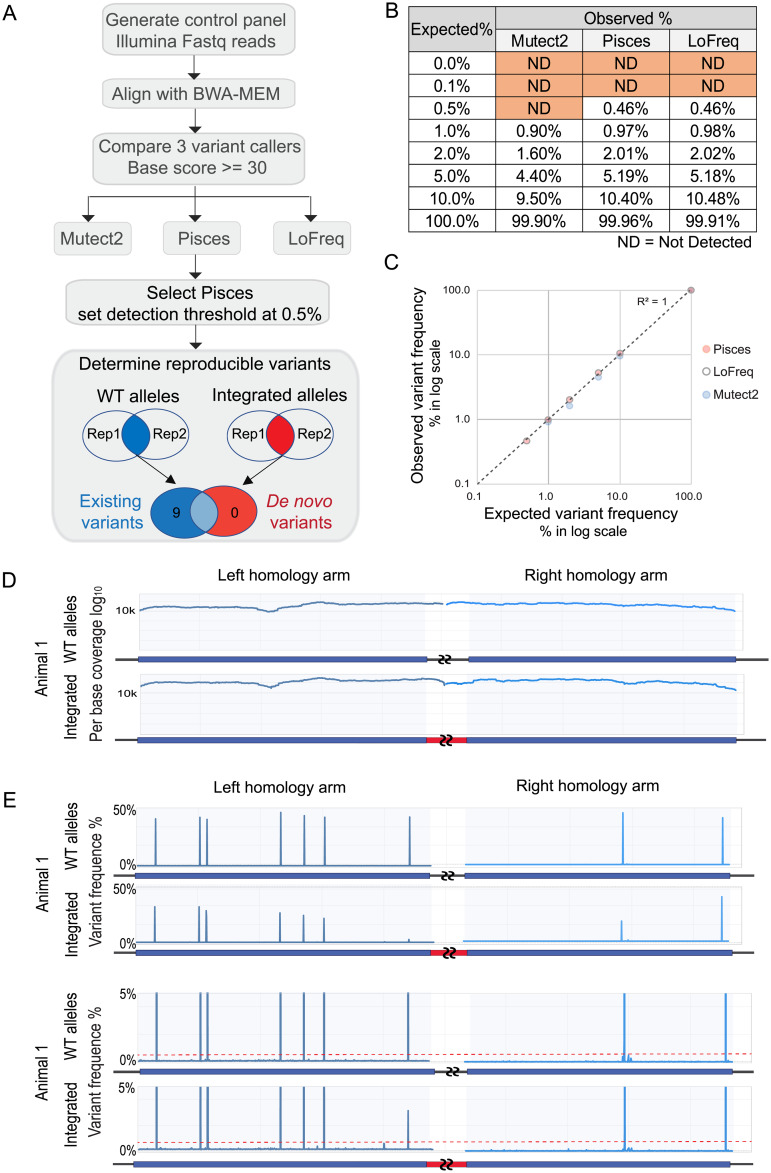
Integration is high fidelity with no *de novo* SNP or indel at homology arms. A. Flow chart of variant calling test. A control panel with known variant frequency was generated and sequenced for testing variant tools. Control fastq data were mapped with BWA-MEM. All three variant calling tools, Mutect2, Pisces, and LoFreq, used the same mapped control bam files as input. Pisces was selected for experimental analysis, with a detection threshold set at 0.5%. Reproducible variants were determined by comparing replicated data sets of WT alleles (unintegrated) or integrated alleles. This step reported 9 existing variants in the WT alleles, and no *de novo* variants in the integrated alleles. B. Performance of three variant calling tools on the control panel. The “Truth” is the known level of SNPs in the control samples, and “Observed” is the level reported from each tool. C. Plotting “Expected” versus “Observed” SNP frequency on a log scale shows a strong linear correlation. The R^2^ for Pisces equals 1. D. Illumina sequencing coverage over left and right homology arms. A representative WT allele sample and integrated sample from animal 1 are shown. Every position on the arms has over 10,000x coverage. Coverage plots for all samples are shown in S2 Fig. E. Variant detection by Pisces. A representative WT allele sample and integrated sample from animal 1 are shown. The Y-axis is capped at 50% variant frequency in the top panel, and 5% in the lower panel. The red dotted line marks 0.5%. Variant analysis for all samples is shown in S2 Fig.

For therapeutic applications of AAV-meditated gene integration, it is important to determine if any unintentional mutations, including SNVs, insertions, or deletions, are introduced at the integrated locus. Over 40 variant calling tools are publicly available. Based on published tool comparisons in the literature, we selected three tools that are suitable for identifying low frequency somatic variants, including single base changes and small indels: Mutect2, Pisces and LoFreq [35–37]. To test the sensitivity of these tools to detect mutations within the genomic region of interest, we generated 8-step standard curve control samples. The controls consisted of right homology arm sequences of hPAH vector genome with a SNV at 0%, 0.1%, 0.5%, 1%, 2%, 5%, 10% and 100% frequencies. These control samples were sequenced using the Illumina MiSeq platform to an average coverage of over 10,000x (i.e., each base was sequenced >10,000 times) that were mapped to the right homology arm reference sequence, and then analyzed using each of the three somatic variant tools (Fig 3A). Fig 3B shows the performance of these tools to detect SNVs across the range of known frequencies. Mutect2 was eliminated due to the low sensitivity at detecting SNVs at 5% or below. Pisces and LoFreq performed equally relative to the expected variant percentage, with a linear correlation between the expected and the observed frequency (Fig 3C). All three programs also reported a number of false-positive variants from the control samples. With the limitation of false positive occurrences within the control samples, anticipated errors associated with PCR and the Illumina platform, we conservatively set our variant reporting threshold at 0.5%.

Integrated and WT alleles from vector-treated human liver genomic DNA purified from two FRG mice were assayed for mutations using previously described allele-specific primers. Technical replicate PCR reactions were sequenced from each test sample to over 10,000x coverage (Fig 3D), and variant detection analysis was performed. The human liver cells were derived from a single human donor, and 9 variants were identified at ~50% frequency in the WT allele sequences (Fig 3E, top panel, y-axis is capped at 50%). All 9 variants were common in the 1000 genomes population with frequencies of 1.2–35%. dbSNP reference numbers are shown [38]. 7 out of 9 SNPs were located within the left homology arm target region and 2 within the right homology arm. In the integrated allele sequences from both animals, these 9 common SNPs were also identified reproducibly at lower frequencies (discussed in the following section).

Importantly, no *de novo* mutations were reproducibly detected (lower limit of detection = 0.5%) in either homology arm target sites in the WT or integrated alleles (Fig 3E, lower panel, y-axis is capped at 5%. S2 Fig for both animals and replicates). These data show that targeted integration of the human PAH cDNA into the *PAH* locus with AAVHSC15 displays high sequence fidelity with no evidence of mutations (LoD = 0.5%). These results support previous observations with other serotypes that AAV-mediated, nuclease-free integration utilizes the HR pathway with no extraneous alterations [5].

### Recombination frequency measured between variants is linear and supports AAV-mediated gene integration by the homologous recombination pathway

**Fig 4:**
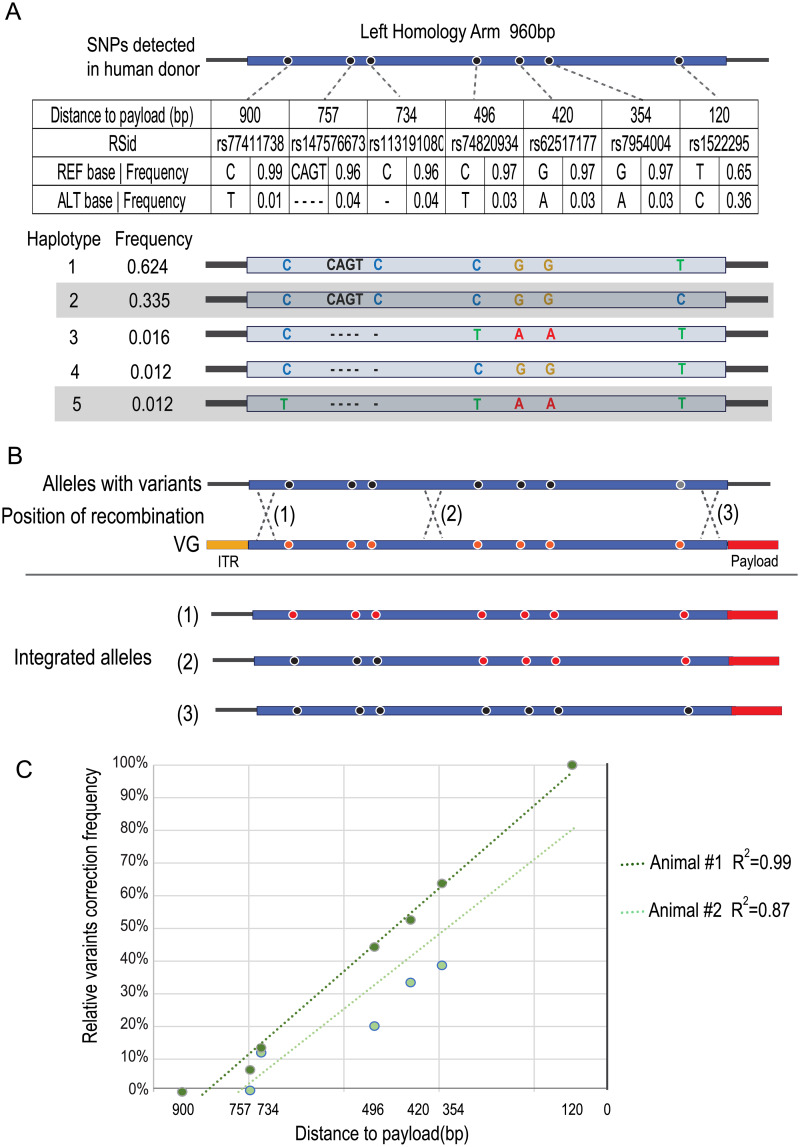
Recombination frequency measured between variants. A. 1000 Genomes haplotypes and frequencies for 7 common variants identified in the left homology arms. The 7 non-reference variants found in the LHA are shown by position, population frequency, and dbSNP number. The five most common haplotypes for this region in the 1000 genomes project are shown with the donor’s two haplotypes shaded. Distance to center indicates the distance to the first base of the payload. B. Illustration of recombination position and variant correction relationship. The variants in the WT, unmodified alleles, are colored in black. These positions in the vector genome are colored in red and are the same sequences as reference genome in hg38. Three scenarios are shown. (1) Recombination occurs at the extreme end of the left homology arm. The resulting integrated allele has all 7 variants “corrected” to the reference sequence by the left homology arm of the vector genome. (2) Recombination occurs in the middle of the arm. Variants toward the payload are corrected. (3) Recombination occurs between the payload and the most proximal variant and no variants are reversed. C. Variant correction frequency plotted against the distance on the left homology arm. A linear trend line is fitted to each sample. Previous work has shown an approximately linear relation between distance and degree of crossover/correction to reference.

The occurrence of non-reference, heterozygous SNPs in the donor genome homology arm region provided an opportunity to assess the location of strand crossovers within the left viral homology arm. The left homology arm is heterozygous at seven positions and six of these are in strong linkage disequilibrium, indicating that these SNPs are likely present on the same allele in the donor genome (Fig 4A) [39]. rs77411738 is the rarest of these SNPs, occurring at a frequency of slightly more than 1% in 1000 Genomes and gnomAD individuals [40, 41]. Notably, this non-reference variant only occurs when variants rs147576673, rs113191080, rs62517177, rs74820934, and rs7954004 are also non-reference and variant rs1522295 is reference. Thus, one donor allele should have only rs1522295 with one variant position as non-reference and the other allele should have six non-reference variants involving a total of 10 positions which represent the second and fifth most common haplotypes in the 1000 genomes populations (Fig 4A). If this level of sequence variation has no impact on the rate of HR, about half of the integration events are likely to occur on the allele with the primarily reference sequence while the other half are likely to occur on the allele with six variants encompassing 10 nucleotides. The deviation of SNP frequency from 50% should provide an estimation of where crossovers have occurred and whether there is any allele specificity (Fig 4B). While not at single-nucleotide resolution, a hot-spot should be recognizable by a sharp change in frequency between a pair of SNPs. As shown in Fig 4C, there is no abrupt change in frequency but, as expected, SNPs further away from the payload are less impacted by the recombination event. This indicates that crossovers must occur at multiple sites within the homology arm. Very few occur in the last 200bp of the homology region [39]. Furthermore, the relatively linear rate of non-reference disappearance indicates there is little or no specificity for the allele in which the integration occurs. If there was specificity against the less identical haplotype, its non-reference variants would not disappear. These findings are consistent with previous work demonstrating AAV-mediated gene integration occurs via the HR pathway [5].

### Integrated alleles do not contain ITRs consistent with HR-mediated integration

A hallmark of HR is that non-homologous sequences such as viral ITRs would lie beyond the extent of recombination and would not be integrated into the target site. If integrated alleles were resolved by HR, the ITR should not be integrated (Fig 4B). However, ITR integration has been reported at sites of NHEJ-mediated integration such as the AAVS1 site and at sites of double-strand breaks [42, 43]. Recent characterization of AAV integration when used in conjunction with a targeted endonuclease identified a high frequency of ITR sequences at endonuclease-generated integration sites, thus introducing on-target mutations with unknown genetic consequences [23]. Therefore, it is critical for AAV-mediated integration platforms to test for the presence of ITR sequences as their detection would strongly suggest that the targeted-integration is resolved through mechanisms such as NHEJ or MMEJ and not HR.

**Fig 5:**
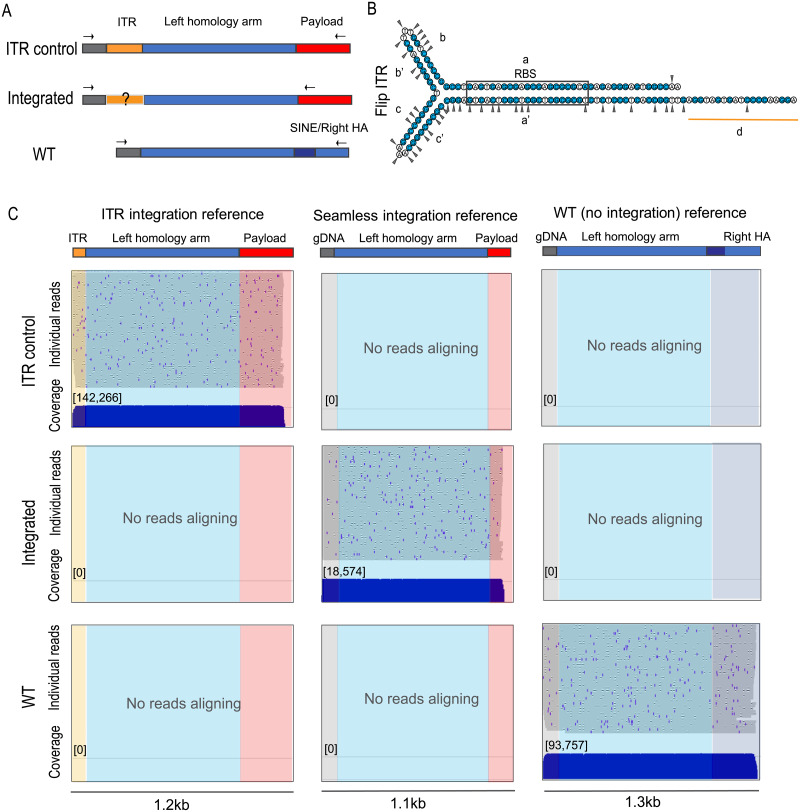
Integrated alleles do not contain ITR. A. Schematic of PCR amplicons for left ITR control sample, integrated allele and WT allele. Note that the reverse primers for ITR control and integrated alleles prime from different locations in the payload. B. ITR sequence in the flip orientation. G and C bases are colored blue. Triangles mark the documented trimming positions at the integration site [6]. C. Oxford Nanopore reads map to a concatenated reference file for left integration site: ITR integration, seamless integration and WT references. Each blue horizontal line represents an individual read and demonstrates the continuity of each long read, total 50 reads. The blue marks and black gaps on the individual reads reflect sequencing errors in each read that occur with Oxford Nanopore technology. The coverage track shows the times the samples were sequenced. From left to right: Reads aligned to “ITR integration reference”, which contains ITR “d region” sequence, left homology arm and partial payload. Reads aligned to “seamless integration reference”, which does not contain ITR, and has a perfect junction between genomic DNA and left homology arm. Reads aligned to “WT (no integration) reference”.

To query for integrated viral ITR sequences within edited loci, the region spanning from payload to genomic DNA outside of the left homology arms was PCR amplified and sequenced using the long-read Oxford Nanopore platform. To satisfy the high input DNA requirement for the library preparation, WT amplicons from animal 1 and 2 were pooled, and integrated amplicons from animal 1 and 2 were pooled. A positive control for ITR integration that reflects a full-length ITR integration between the end of the left homology arm and genome DNA was generated by PCR (Fig 5A, showing Left integration).

Several studies have systematically characterized the ITR sequences that integrate into the host genomes [6, 44]. These studies suggest that ITR sequences were processed and parts of the ITR were trimmed off during integration so that only the untrimmed portions of the ITR were recovered at the integration sites (Fig 5B, triangles mark documented trimming positions in [6]). Therefore, we focused the analysis on the relatively stable end of the ITR, the “d region” (underlined in Fig 5B). Nanopore reads, derived from genomic DNA from both treated animals, spanning the integration site with sequence quality score above 90 were mapped to a concatenated reference containing three parts: (1) integration of the ITR “d region”into the *PAH* locus, (2) seamless integration into the *PAH* locus without any trace of ITR, and (3) the WT reference. For each sample, a random subset of 50 reads are displayed as individual reads where each horizontal line represents a single long sequencing read that covers the payload or WT sequence, homology arm and either the genome or the ITR.

The compiled coverage shows that each sample was sequenced deeply (over 10,000 reads, Fig 5C. Right integration shown in S3 Fig). The ITR control reads mapped to the ITR integration reference showing this assay can detect ITR integration. WT alleles had no reads with ITR sequence. Analysis of integrated alleles from AAVHSC-treated animals showed seamless integration on both left and right sides. Zero out of 18,574 and 0 out of 100,588 reads mapped to the left and right ITR integration references, respectively. All together, these data show that the integrated alleles are free of ITR sequence, consistent with HR as the main mechanism for hPAH vector-mediated integration.

## Discussion

Genome editing technologies are rapidly advancing into the clinic, and the changes they introduce can be maintained and propagated stably during the life of the target cells [15]. As these technologies are being developed to treat human disease, the molecular consequences at the specific gene editing site require accurate quantitation of editing frequency and the determination of any undesired mutations.

Currently there is a lack of standardized approaches for measuring AAV-mediated integration frequency at the DNA level as well as for the precision of the changes being introduced. We report here precise *in vivo* insertion of the full human PAH cDNA into intron 1 of the *PAH* locus in human hepatocytes to a level of roughly 6%. This measurement was accomplished using two orthogonal methods that both assessed DNA directly rather than indirect expression or protein measurements. This integration rate, observed at a high dose of 1e14 vg/kg in the humanized FRG model, is an improvement in efficiency relative to many previous studies [21, 45, 46]. However, at present, such cross-study comparisons are difficult due to the inconsistency of measurement methods used, including the lack of characterization at the DNA level. A number of observations of AAV-mediated targeted integration has been made using *in vitro* cell culture systems. These are often in immortalized cells with aneuploid genomes and unknown mutation profiles, which might greatly impact DNA damage response mechanisms. In addition, it is well-accepted that the *in vitro* transduction efficiency of AAVs does not translate to *in vivo* systems. Thus, *in vitro* studies are of limited use for the characterization of AAV biology for potential *in vivo* therapeutic use in humans [47]. With this in mind, we employed the humanized FRG model to enable the testing of these vectors in as relevant a model as is available, functional human hepatocytes. While the dose of 1 x 10^14^ vg/kg is a significantly higher dose compared to current AAV-mediated gene therapies in the clinic targeting the liver, AAV mediated integration into the genome via homologous recombination will likely require higher concentrations of vector [48]. In general, the immune response to AAV therapies does correlate with dose, with the most common adverse event being transient elevations in liver enzymes that are responsive to corticosteroids. There are several ongoing clinical trials specifically targeting the liver using AAV-mediated gene therapies that range in doses from the low 10^13^ vg/kg for OTC (Clinical trial identifier NCT02991144) to 6x10^13^ vg/kg for hemophilia A (NCT03370913). A similar dose of AAVHSC15 (0.7x10^14^vg/kg injection) caused no adverse effects in non-human primates [27]. In addition, systemic doses of AAV in the 1x10^14^vg/kg range are being used clinically for indications targeting the CNS and muscle. While not liver directed, the FDA recently approved a systemic AAV treatment using 1.1 x 10^14^vg/kg for spinal muscular atrophy (SMA) (BLA STN#: 125694/0), and at least two systemic AAV clinical trials are currently using doses from 1x10^14^ to 3x10^14^ vg/kg (Clinical trial identifier: NCT03333590, NCT03199469).

There are limitations even with this murine model. The proliferation rate of the donor human hepatocytes in FRG mice is higher than that of a human liver. This increase in cellular division, coupled with the 1x10^14^ vg/kg dose, may impact the rate of homologous recombination, particularly when NTBC is removed to allow the selective growth and repopulation of the human hepatocytes, contributing to the increased levels described here. However, as discussed by Ginn et al this may be more representative of the active hepatocyte cellular division occurring during early child development, the target population for many genetic medicine therapeutics [49]. In an attempt to limit the impact of active proliferation, we focused the studies at a timepoint when the liver repopulation had reached a plateau of roughly 80% human hepatocytes. While this murine model has limitations, the data obtained in this *in vivo* humanized system can be used as a guide to characterize the precision and accuracy of the integration in functioning human hepatocytes as part of the preclinical development of novel gene integration therapies. Both mouse and human hepatocytes in the FRG chimeric mice have functional copies of the *PAH* gene and do not exhibit PKU phenotypes. Thus, this model cannot be studied for phenotypic correction of PKU, but rather it is a model to assess the integration of cDNA into specific loci of the human genome in an *in vivo* context. We used FRG mice as the closest surrogate to characterize transduction, editing and fidelity of AAV-guided gene integration in functional human hepatocytes.

There are a variety of methods used in analytical characterization of AAV-mediated integration including DNA analysis by TOPO cloned PCR amplicons and counting colonies and short amplicon NGS sequencing. Other methods do not interrogate editing at the DNA level but rather use promoter-driven reporter gene expression such as GFP as a proxy of integration frequency [12, 21, 23, 48, 50–52]. While expression of protein is an important readout of the effectiveness of the vectors, caution should be taken to equate expression with DNA editing. Editing vectors often contain sequences that can contribute to transcriptional activity, especially when the targeted integration site is proximal to an endogenous promoter, such as the hPAH vector in this study. In addition, the AAV2 ITR sequence used in most editing vectors has slight promoter activity that can drive expression without integration [53, 54]. Many of these methods do not capture the potential risk of *de novo* mutations at the editing target or distinguish between HR and NHEJ insertion.

Measurement of targeted gene integration by an AAV-delivered repair template must overcome technical obstacles such as excess episomal vector genomes and lengthy homology arms. The 3-primer NGS assay described here quantifies integration efficiency covering the extent of the integration site. This assay corrects for PCR efficiency between each genotype-specific amplicon and is linear across a range of detection from 0.5% to 10% of integrated alleles. In addition, with proper primer selection and sample preparation, this assay can tolerate the presence of excess repair templates such as episomal vector genomes. An important limitation of this approach is the dependence on accurate measurement of PCR efficiency, necessitating careful empirical testing of primers and conditions. Further, PCR efficiency of control amplicon templates may be different than genomic templates. These limitations may be overcome by the generation of an isogenic cell line in which the frequency of the integrated allele is known.

Previous reports have shown that AAV-mediated target integration can be precise. However, this has not been tested at the DNA level in all of these studies, thus making it difficult to directly compare across studies [12, 50, 52]. To characterize the precision of AAVHSC15-mediated gene integration, we employed Illumina sequencing to analyze the sequence across each homology arm of the integrated alleles. Sequence coverage of >10,000 reads spanning each homology arm enabled the determination of the on-target mutation profile. We analyzed positive control sequences using three commonly employed variant callers to determine the lower limit of detection and the false positive rate for each across an allelic range of frequencies from 0.1% - 99%. Based on this analysis, we selected the Pisces variant caller and set the lower level detection limit at 0.5% for analysis of these AAVHSC15-treated samples. The resulting variant detection analysis shows that no *de novo* mutations were introduced apart from the desired targeted gene integration at or above the lower limit of detection. We note that as these methods employ site-specific PCR, this assay will not detect structural variants such as large rearrangements and translocations.

One of the outcomes of homology-directed repair is the crossover between homologous sequences. The approximate location of strand crossover can be determined by changes in sequence variants that distinguish the homologous donor template from the target sequence. The quantitative measurement of inherited variants across the WT and integrated alleles identified 7 SNPs in the region homologous to the left arm and 2 in the right arm. As these variants are in the NCBI dbSNP database and were present in both the integrated and WT sequences, they must be part of the genetic background of the human hepatocyte donor. Each of the variant alleles is present at approximately 50% frequency in the WT amplicons, indicating these variants are heterozygous in the human donor liver cells. Based on gnomAD and 1000 Genome haplotype frequencies, 6 of the 7 variants in the left homology arm are on the same allele [38, 40, 41]. Quantitative variant calling enabled us to use the allele frequency changes in the integrated amplicons to gain insight into the relative location of the homology arm crossovers. Analysis of these data supported the mechanism of homologous recombination occurring at multiple sites along the homology region with no particular hotspots. Furthermore, the near complete disappearance of some of the variants shows that both alleles are subject to integration and the rate of disappearance indicates that there is little or no difference in the rate of integration even though there are 10 positions that vary from reference in one of the alleles.

We also described a long-read sequencing method to capture the entire sequence from the inserted payload through the homology arm and into the native genome to test for the presence of viral ITR integrations. As ITR sequences are highly structured, PCR amplification and subsequent sequencing through them is challenging. Oxford Nanopore sequencing of control templates with an integrated ITR shows this method can detect viral sequence when present. The majority of reads from ITR-containing controls showed contiguous sequence from the payload through the homology arm and into the d region of the ITR. No ITRs were detected in the integrated alleles. A potential limitation of this assay is that the highly structured nature of ITRs introduce a risk of being undercounted but the single-molecule nature of Oxford Nanopore minimizes that risk.

## Conclusions

If AAV-mediated, non-nuclease *in vivo* target integration is to be advanced as a novel therapeutic modality, it is necessary to include characterization of the efficiency and the nature of the changes being introduced to the target genome at the molecular level. Here we described a novel framework for assessing *in vivo* AAV-mediated targeted integration events in human hepatocytes using two orthogonal methods to measure on-target integration frequencies: one method to detect *de novo* mutations, and another method to query ITR integrations. Although these methods require optimization to target different genomic loci, developing these molecular methods should be applied to fully characterize *in vivo* AAV-mediated target integration. Using the hPAH vector as an example, we demonstrated that this vector design achieved non-nuclease mediated gene integration at a frequency of 6%, without *de novo* mutation or ITR integration at or above a lower limit of detection of 0.5%. Altogether, the absence of *de novo* mutation, the linear pattern of somatic SNP reversal, and no detectable ITR integration aligns with previous findings demonstrating homologous recombination as the mechanism of AAV-mediated, targeted integration. We believe that these molecular methods provide a framework for researchers to characterize targeted integration, or editing events, and assure greater precision in molecular characterization and comparability of data, which may lead to further advances in AAV-mediated HR gene editing technologies.

## Supporting information

S1 Fig Control panels for 3 primer NGS. Five step editing standards composed of 0%, 1%, 2%, 5% and 10% of edited alleles (left or right) were PCR amplified, sequenced and analyzed as testing samples. The standard curves were plotted as “Observed integration efficiency” vs. “Anticipated”. The trendlines for both left and right integration sites were fitted and calculated the correction formulas. The formulas were applied to the testing samples to calculate “corrected” editing efficiency.
(EPS)

S2 Fig Sequencing coverage profile and variant detection for both animals. A. Illumina sequencing coverage over left and right homology arms. Every position on the arms has over 10,000 coverage. There was unknown source of sample contamination in animal 2, Integrated alleles, right side, rep2, and this sample was excluded from variant analysis. B. Variant detection by Pisces. Variant analysis for all samples is shown. Y-axis is capped at 5% variant frequency. The red dotted line marks 0.5%. There are several positions with SNP frequency over 0.5% in animal 2, integrated alleles, left side, rep1. None of these positions are reproducibly detected in the other technical replicate, they are not reported as *de novo* mutations with our criteria described in the main text (Fig 3A).
(EPS)

S3 Fig Integrated alleles do not contain ITR on the right integration site. Nanopore reads map to a concatenated reference file for Right integration site: ITR integration, seamless integration and WT references. Each blue horizontal line represents an individual read and demonstrates the continuity of each long read, total 50 reads. The coverage track shows the times the samples were sequenced. From left to right: Reads aligned to “ITR integration reference”, which contains ITR “d region” sequence, left homology arm and partial payload. Reads aligned to “seamless integration reference”, which does not contain ITR, and has a perfect junction between genomic DNA and left homology arm. Reads aligned to “WT (no integration) reference”.
(EPS)

S1 Table Primer and probe sequences. (DOCX)

S1 Raw images (PDF)

## Abbreviations

AAV: adeno-associated virus
AAVHSC: adeno-associated virus derived from hematopoietic stem cell
DSB: double-strand break
HR: homologous recombination
ITR: inverted terminal repeat
NGS: next generation sequencing
NHEJ: non-homologous end joining
PAH: phenylalanine hydroxylase
PKU: phenylketonuria
SNP: single nucleotide polymorphism
SNV: single nucleotide variant
