# Supplementary material for: Molecular characterization of precise in vivo targeted gene integration in human cells using AAVHSC15
Source: PLoS One. 2020 May 26;15(5):e0233373. doi: 10.1371/journal.pone.0233373 (PMC7250422; doi:10.1371/journal.pone.0233373)
Supplement: S1 Table — (DOCX) [file pone.0233373.s004.docx]

| **Fig 1. TI PCR** |  |
| --- | --- |
| Left integration site. PCR round 1 |  |
| L.gDNA.F2.5 | GACTAGAACACCACCAAGCACA |
| CoPAH.5'.R | AACTCGTACTCGTCCTTCTTCAGT |
| Left integration site. Nested PCR |  |
| L.gDNA.F3 | CCTATTCTTAAAACCTTCCAGCAA |
| 2A.R | AGACTTCCTCTGCCCTCTCC |
| Right integration site. PCR round 1 |  |
| 1000.F1 | CCTAACCCTCTCCTCGGTCT |
| R.gDNA.R2 | AACTTAGAGCCAAAGGGAGAAAAT |
| Right integration site. Nested PCR |  |
| 1000.F1 | CCTAACCCTCTCCTCGGTCT |
| R.gDNA.R3 | CCATCCCAGGTGATTCTTAACCATAG |
| **Fig 2. Linkage ddPCR** |  |
| CO-PAH payload primers and probe |  |
| CO-PAH-FWD Set 2 | GTATTATCCAGCACCTCGATCC |
| CO-PAH-REV Set 2 | GTACTATGTGGCCGAGTCTTT |
| CO-PAH PRB Set 2 | /56-FAM/ACGATGCCA/ZEN/AGGAGAAGGTGAGAA/3IABkFQ/ |
| Human PAH genomic primer and probe |  |
| hPAH_gDNA FWD Set 2 | AGTGCATGGAGAGAAATGGAG |
| hPAH_gDNA REV Set 2 | CACCAGACAGTTAGTCAATAGCA |
| hPAH_gDNA PRB Set 2 | /5HEX/ACAGCCTAT/ZEN/ATTTCACCATGCTGATCCC/3IABkFQ/ |
| Mouse Pah genomic primer and probe |  |
| mPah_gDNA FWD Set 1 | CAGCATCAGAAGCAGAACATTT |
| mPah_gDNA REV Set 1 | AAAGCACATCAGCAGTTTCAA |
| mPah_gDNA PRB Set 1 | /5HEX/AGATGAAAG/Zen/CAACTGAACATCGACTACGA/3IABkFQ/ |
| **Fig 2. 3 primer NGS** |  |
| Left integration site |  |
| L.gDNA.F3 (shared) | CCTATTCTTAAAACCTTCCAGCAA |
| RHA_5’R (WT specific) | CCAGGGCTAACTCTTGGTCA |
| CoPAH.5'.R (integrated specific) | AACTCGTACTCGTCCTTCTTCAGT |
| Right integration site |  |
| **Fig 3. Fidelity assay** |  |
| WT, Left homology arm |  |
| L.gDNA.F3 | CCTATTCTTAAAACCTTCCAGCAA |
| RHA_5’R | CCAGGGCTAACTCTTGGTCA |
| WT, Right homology arm |  |
| R.gDNA.R3 | CCATCCCAGGTGATTCTTAACCATAG |
| LHA_CtoTF | CAGAGAAAGAGATCCGAAGACTGCTGGTG |

**S1 Table. Primer and probe sequences**
