# Supplementary material for: Molecular characterization of precise in vivo targeted gene integration in human cells using AAVHSC15
Source: PLoS One. 2020 May 26;15(5):e0233373. doi: 10.1371/journal.pone.0233373 (PMC7250422; doi:10.1371/journal.pone.0233373)
Supplement: S1 Raw images — (PDF) [file pone.0233373.s005.pdf]

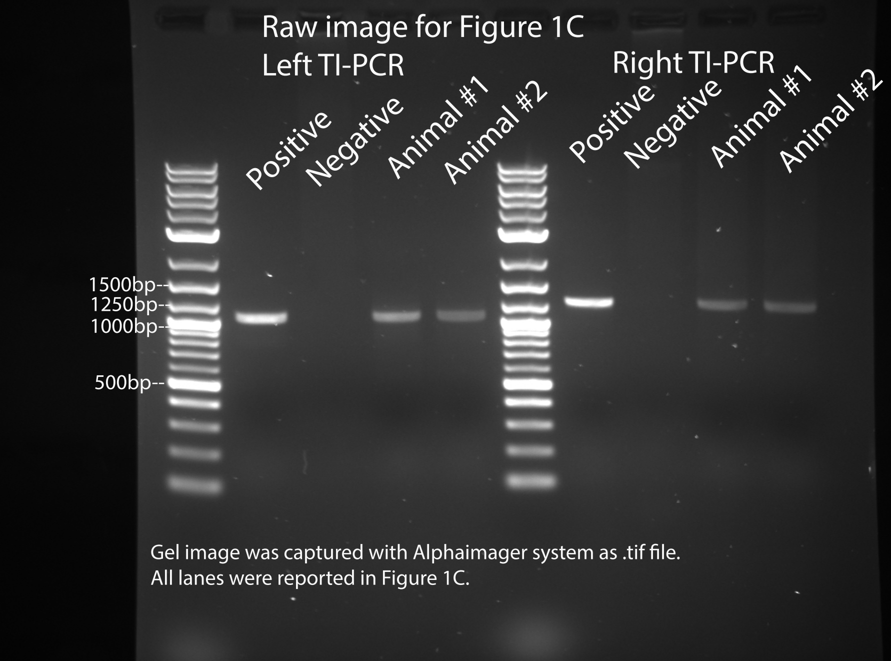
Raw image for Figure 1C
Left TI-PCR
Right TI-PCR
Positive
Negative
Animal #1
Animal #2
Positive
Negative
Animal #1
Animal #2
1500bp--
1250bp--
1000bp--
500bp--
Gel image was captured with Alphaimager system as .tif file.
All lanes were reported in Figure 1C.
